# The Role of Immune Infiltration and Oxidative Stress in the Progression of Cerebral Cavernous Malformation

**DOI:** 10.1002/brb3.71237

**Published:** 2026-01-31

**Authors:** Xuesai Zhu, Yizhi Yao, Tengbo Yu, Xiao Xiao

**Affiliations:** ^1^ Central Laboratories Qingdao Municipal Hospital, University of Health and Rehabilitation Sciences Qingdao Shandong China; ^2^ Department of Orthopedic Surgery, Key Laboratory of Orthopedics, Sports Medicine & Rehabilitation, Qingdao Municipal Hospital University of Health and Rehabilitation Sciences Qingdao Shandong China

**Keywords:** cerebral cavernous malformation, immune microenvironment, inflammatory response, pathogenesis, oxidative stress

## Abstract

**Purpose of Review:**

To review how the immune microenvironment and oxidative stress modulate the initiation, maturation, and hemorrhagic conversion of cerebral cavernous malformations (CCM) and to appraise the therapeutic potential of immune‐directed interventions.

**Finding:**

This project conducts systematic research on the pathogenesis of cerebral cavernous malformation (CCM), focusing on the complex interactions between genetic mutations (KRIT1, CCM2, PDCD10) and the immune microenvironment, oxidative stress, inflammatory responses, and vascular dysfunction. The study confirms that CCM development relies not only on genetic mutations but also on the synergistic effects of a “second hit” mechanism and microenvironmental stressors. Immune cell infiltration (e.g., B cells, T cells, neutrophils) and oxidative stress responses play pivotal roles in lesion progression; Blood‐brain barrier disruption and immune thrombosis further exacerbate the pathological process. These findings provide theoretical foundations for understanding CCM's multifactorial pathogenic network and establish scientific bases for personalized therapeutic strategies targeting the immune microenvironment and oxidative stress.

**Conclusion:**

Genetic mutations trigger the formation of initial CCM lesions, while environmental and immune factors promote disease progression, increasing the risk of abnormal cerebral vascular formation or rupture.

## Graphical abstract

Mechanism of rupture hemorrhage in cerebral cavernous malformations. Environmental and immune factors promote the progression of lesions and induce the risk of abnormal formation or rupture of cerebrovascular vessels.

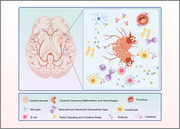

## 1 Introduction

Cerebral Cavernous Malformation (CCM), also known as cerebral cavernous angioma, is a type of central nervous system vascular malformation characterized by angiographic insidiousness and hyperemia (Zhang et al. 2024), with a population prevalence estimated to be between 0.3% and 0.9% (Al‐Holou et al. 2012; Akers et al. 2017). Anatomically, CCM lesions are composed of abnormally dilated, thin‐walled vascular sinusoids lined by a single layer of endothelial cells (ECs), with absent or dysfunctional tight junctions between these cells (Zhang et al. 2024; Valentino et al. 2024; Snellings et al. 2021). At the microscopic level, abnormal endothelial cell proliferation and disruption of tight junction structures are seen, leading to significant vascular leakage (He et al. 2024). Although CCM can occur at multiple sites throughout the body, its striking clinical signs (e.g., focal neurologic deficits, seizures, etc.) are seen primarily in brain and spinal cord lesions (He et al. 2024; Al‐Shahi Salman et al., 2012; Flemming 2017; Flemming and Lanzino 2020).

Studies on the pathogenesis of CCM have progressed over decades, and it is now clear that familial or sporadic mutations are the molecular basis (Zafar et al. 2019; Hong et al. 2021; Weng et al. 2021). CCM can present as an autosomal dominant hereditary disease (OMIM116860), with approximately 20–30% of cases being familial, while the remaining 70–80% are sporadic (which can manifest as solitary lesions or be associated with developmental venous anomalies) (Akers et al. 2017; Al‐Shahi Salman et al., 2012). The conventional view is that CCM is congenital, and some lesions may remain biologically quiescent for long periods of time; however, continuous MRI monitoring confirms the presence of a dynamic process of lesion neogenesis, volume increase, and recurrent hemorrhage (Acciarri et al. 2009; Yadla et al. 2010). Notably, genetic factors cannot fully explain the heterogeneity of the clinical behavior of the lesions. The fact that CCM injury does not occur with the induced knockout of specific genes after vascular development suggests that the temporal window of genetic change and possibly the resulting specific changes in the microvascular environment may be essential for the CCM phenotype (Boulday et al. 2011). Loss of CCM protein function is involved in pathogenesis through pleiotropic mechanisms that regulate angiogenesis, vascular homeostasis, and the cellular stress response (Marchi et al. 2016). The immune microenvironment plays an important role in the progression of a variety of diseases, and in recent studies it has been found to play a key role in the development and progression of cavernous vascular malformations (Li et al. 2023).

In recent years, tremendous progress has been made in understanding the pathogenesis of CCM disease, revealing a striking complexity (Marchi et al. 2016). However, of even greater importance is how this complexity is understood and how to account for the fact that interrelated mechanisms can combine to influence the onset, progression, and severity of CCM disease. A growing body of in vitro and in vivo evidence suggests that oxidative stress, inflammatory responses, and dysregulated angiogenesis are key components driving CCM pathology (Zhu et al. 2010; Shi et al. 2016; Goitre et al. 2010; Wüstehube et al. 2010; Choquet et al. 2014). The aim of this article is to provide a systematic review of the CCM immune microenvironment and the impact of oxidative stress on lesion progression and to explore therapeutic perspectives for targeting the immune microenvironment.

## 2 Molecular Basis and Mechanisms of CCM

**FIGURE 1:**
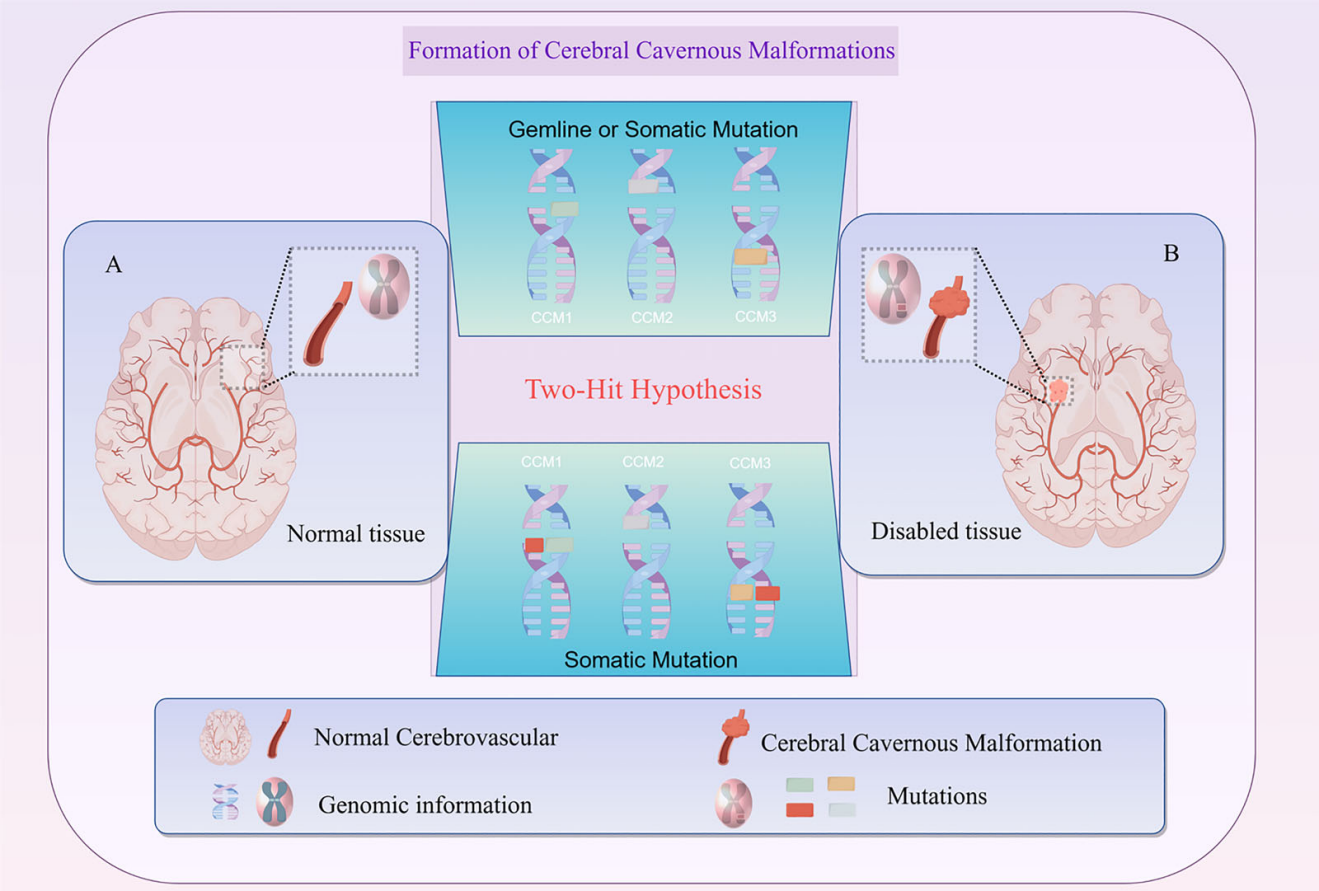
Genetic basis and the “two‐hit” mechanism in Cerebral Cavernous Malformation (CCM). (A) Schematic representation of normal cerebrovascular tissue without CCM‐associated mutations. (B) Lesion genesis via a germline mutation followed by a somatic “second hit” in endothelial cells, leading to complete loss of CCM protein function.

Despite the fact that genetic studies have identified KRIT1 (CCM1), MGC4607 (CCM2), and PDCD10 (CCM3) as the major pathogenic genes for CCM (Choquet et al. 2015; Riant et al. 2010), current evidence indicates that a single gene mutation alone is not sufficient to drive the occurrence and progression of CCM. The pathological process relies on a two‐hit mechanism and the synergistic action of microenvironmental stress factors, including oxidative stress, inflammatory interference, and factors affecting endothelial cell homeostasis (Choquet et al. 2015; Riant et al. 2010; Retta and Glading 2016) (Figure 1).

Histological analyses suggest that the development of CCM lesions requires the complete loss of CCM gene function through a two‐hit molecular mechanism (McDonald et al. 2011; Akers et al. 2009). CCM proteins are not only associated with basic physiological functions but also involved in regulating various redox‐sensitive signaling pathways and mechanisms, including pro‐oxidant and antioxidant pathways as well as autophagy. CCM proteins not only regulate the stability of endothelial cell junctions and the formation of vascular lumens but also participate in pathological processes by modulating redox‐sensitive pathways. (Marchi et al. 2016; Goitre et al. 2010; Marchi et al. 2015).

Hypoxia is a potent driver of angiogenesis and thrombosis in cardiovascular disease (Gupta et al. 2029). A slight reduction in oxygen levels can significantly increase angiogenesis, neuroinflammation, and thrombosis in CCM disease by enhancing cell‐cell interactions. A slight reduction in oxygen levels can significantly increase angiogenesis, neuroinflammation, and thrombosis in CCM disease by enhancing interactions between endothelial cells, astrocytes, and immune cells, revealing hypoxia as a critical environmental factor driving variations in CCM severity (Gupta et al. 2017; Rius et al. 2008; Frias‐Anaya et al. 2024; Fong 2008; Eltzschig and Carmeliet 2011).

Animal models further reveal that the inherent vulnerability of cerebral vascular endothelial cells makes them susceptible to local stressors (such as changes in blood flow shear force, infection, or trauma), thereby accelerating CCM progression (Leblanc et al. 2009; Whitehead et al. 2009).

**FIGURE 2:**
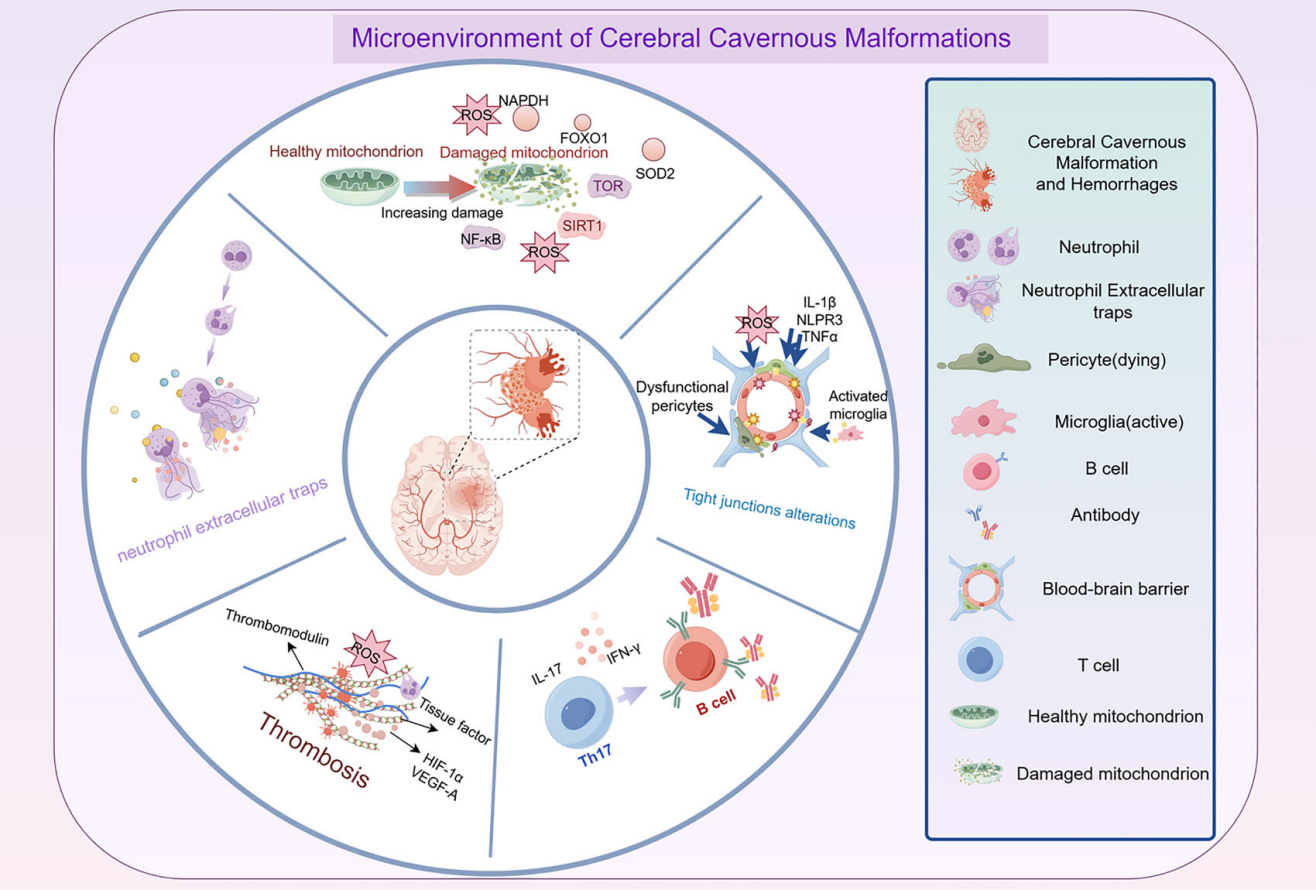
The dysregulated immune and oxidative stress microenvironment drives CCM progression. This schematic integrates key pathological processes driving CCM progression and hemorrhage. Central to the figure is the established CCM lesion. This schematic integrates key processes: activation of microglia/pericytes; neutrophil infiltration and NETosis; B‐cell expansion and autoantibody production; T‐cell recruitment and pro‐inflammatory cytokine release; and mitochondrial dysfunction and oxidative stress.

Currently, CCM is regarded as the result of the intersection of many factors, forming a complex disease pathological network with the involvement of factors such as immunity, hemodynamics, and angiogenesis (Figure 2) (Snellings et al. 2021; Rustenhoven et al. 2021).

## 3 CCM Gene Mutations Originate Within Endothelial Cells

CCM is a disease where endothelial cell homeostasis is disrupted, resulting in increased cellular activation (Onyeogaziri et al. 2024). The pathogenesis of CCM is initiated by endothelial cells (ECs) harboring mutations in CCM‐associated genes. Through clonal expansion, these mutated ECs form a dysplastic vascular bed. As the lesion enlarges, the cells up‐regulate stem‐cell markers that recruit neighboring wild‐type ECs, thereby fueling CCM growth and driving disease progression (Malinverno and Maderna 2019; Detter et al. 2018). CCM proteins are integral to the maintenance of the endothelial barrier. The Rho‐ROCK axis perturbs barrier integrity by governing the expression and subcellular localization of the tight‐junction proteins occludin and claudin‐5; notably, ROCK directly phosphorylates both occludin and claudin‐5, culminating in blood‐brain barrier dysfunction (Yamamoto et al. 2008).

The MEKK3/KLF4 axis operates as a central hub governing CCM initiation and progression (Boulday et al. 2009; Chan et al. 2011). Vascular Endothelial Growth Factor (VEGF) potentiates endothelial injury and angiogenesis (Lopez‐Ramirez et al., 2010), whereas loss‐of‐function of any CCM gene ignites the MEKK3‐MEk5‐ERK5‐MEF2 cascade, provoking a robust KLF4 up‐regulation in ECs (Maddaluno et al. 2013). Elevated KLF4, in turn, boosts endothelial nitric‐oxide (NO) production, which stimulates astrocytic VEGF secretion and thereby forges a self‐reinforcing loop between CCM‐affected ECs and astrocytes that fuels vascular lesion expansion (Min and Zhou 2023; Li et al. 2024). Downstream of CCM mutations, RhoA/ROCK signaling is persistently activated (Ayata et al. 2024) heightened RhoA/ROCK activity drives stress‐fiber assembly, destabilizes inter‐endothelial tight junctions, and orchestrates an EC phenotypic switch characterized by enhanced migration, cell‐cycle arrest, augmented extracellular‐matrix (ECM) breakdown, leukocyte chemotaxis, and inflammatory activation (Ayata et al. 2024; Dulamea and Lupescu 2024).

Animal model studies further elucidate the regulatory mechanisms of ECs in CCM: In mouse models, EC inactivation of Krit1 or Pdcd10 genes leads to elevated expression levels of thrombomodulin (TM) and endothelial protein C receptor (EPCR), while simultaneously promoting EC production of activated protein C (APC) (Wang et al. 2020; Valentino et al. 2020). Mechanistically, KRIT1 or PDCD10 deficiency induces KLF2/KLF4 upregulation, which is a key driver of increased TM expression and ultimately exacerbates the hemorrhagic tendency in CCM lesions (Wang et al. 2020; Valentino et al. 2020). In chronic CCM disease models, chronic neuroinflammation drives epigenetic reprogramming of brain EC subtypes (Gallego‐Gutierrez et al. 2025). Furthermore, EC behavior and differential gene expression profiles between arterial and venous ECs are significantly altered in CCM: EC cytoskeletal remodeling induced by CCM signaling defects enhances cell motility, manifesting as flattening of venous ECs in the microvascular system leading to microvascular dilation, while arterial ECs exhibit inward migration and restricted lumen formation (Yang et al. 2023; Chen et al. 2024).

## 4 Inflammatory Responses Promote the Progression of CCM

Neuroinflammation is an important defense mechanism of the central nervous system, but it is pathologically activated in CCM (Lai et al. 2022). There is a large number of immune cell infiltrations in CCM lesions, including macrophages, microglia, T cells, and B cells (Li et al. 2023). and the degree of infiltration is related to the invasiveness of the lesion and the risk of recurrence (de Souza et al. 2019). It is worth noting that early prelesions carry CCM gene mutations but lack bleeding and immune infiltration, suggesting that the immune response may be secondary (McDonald et al. 2011; Akers et al. 2009).

### 4.1 The Core Role of Immune Infiltration and B Cells

Histological studies have shown that B cell infiltration exists in both quiescent CCM lesions and invasive lesions (characterized by new bleeding) (Shi et al. 2009; Shi et al. 2007). The IgG response is dominant and is not related to the recent clinical activity status. At the same time, lymphocytes expressing IgM and/or IgA are occasionally found in several lesions (Shi et al. 2009). These immune cells target cytoskeletal elements that are usually present in endothelial cells and astrocytes, including vimentin, myosin, and tubulin (Zhang et al. 2020). An antigen‐directed oligoclonal IgG immune response exists within the lesion, regardless of genotype or recent clinical activity (Shi et al. 2009). Although it is believed that immunoglobulins and other related genes are not significantly regulated in human CCM lesions (Shenkar et al. 2003). In situ B cell clonal expansion and antigen‐driven affinity maturation occur in CCM (Shi et al. 2014).

A transcriptomic analysis of acute and chronic CCM3 mouse lesion models revealed an enrichment of adaptive immune response pathways in the chronic model, such as neutrophil chemotaxis and activation, chemokine production, and cytokine secretion (Koskimäki et al. 2019). The transcriptome of the acute CCM3 model is primarily enriched in angiogenesis and cell proliferation pathways (Koskimäki et al. 2019). Compared with non‐recent hemorrhage, the transcriptome of CCM patients with recent hemorrhage shows several enriched pathways associated with acute inflammation, indicating that inflammation may drive hemorrhagic susceptibility (Lyne et al. 2019). In vivo studies in mice have confirmed that B cells are a key factor in the maturation of CCM lesions into clinically relevant phenotypes. B‐cell depletion does not affect the initial lesion occurrence but prevents the maturation and progression of the expanding vessels (Shi et al. 2016). Selecting anti‐BR3 antibodies as B‐cell depletion agents can significantly reduce mature CM and significantly decrease hemorrhagic iron deposits in lesions (Shi et al. 2016). TGFβ may be a key participant in the mechanisms of CCM disease severity and phenotypic variability. Inhibiting TGF‐β signaling can reduce vascular leakage and lesion burden in CCM1‐deficient models (Maddaluno et al. 2013).

### 4.2 Neutrophils: The Bridge Connecting Innate and Adaptive Immunity

Neutrophils in CCM have been proven to play a role in the occurrence of inflammatory responses and the induction of other diseases, such as intracerebral hemorrhage (ICH), cerebral arteriovenous malformations, and cancer (Lai et al. 2022; Olsson and Cedervall 2016). Other researchers have associated the immune response in CCM and other cerebrovascular abnormalities with the lymphatic system and cerebrospinal fluid flow (Rustenhoven et al. 2021).

In CCM, neutrophils drive a vicious cycle through NETosis (neutrophil extracellular traps). NETosis markers (cfDNA/MPO‐DNA complexes) are elevated in the plasma of CCM patients and are associated with recent hemorrhage (Yau et al. 2022). NET formation, also known as NETosis, is considered an innate immune function of neutrophils (Yang et al. 2019) and can be triggered by pro‐inflammatory cytokines (IL‐1β/TNF‐α) as well as activated platelets and endothelial cells (Lai et al. 2022, Gupta et al. 2010). Due to their cytotoxic effects, excessive NETosis can lead to further endothelial damage (Yau et al. 2022, Villanueva et al. 2011).

NETs can activate T/B cells through autoantigen presentation, mediating adaptive immunity (Dömer et al. 2021, Carmona‐Rivera et al. 2020). The formation and release of NETs also depend on the production of reactive oxygen species (ROS), intersecting with oxidative stress in CCM and playing a significant role in its pathogenesis (Onyeogaziri et al. 2024; Kelly et al. 2008).

### 4.3 Th17 Cells Activate Infiltrating Immune Cells

It is noteworthy that the composition (heterogeneity) of inflammatory cell populations in the lesion microenvironment is associated with disease phenotypic characteristics or clinical outcomes. In CCM, sporadic CCM exhibits more pronounced T‐cell infiltration than familial CCM (Shi et al. 2009; Castro et al. 2022). A higher ratio of Th17 to Treg is observed in CCM lesions, which is associated with a clinical phenotype dominated by pro‐inflammatory T cells (Castro et al. 2022). The increased frequency of Th17 cells (typically expressing CCR6) is significantly associated with the clinical activity of CCM, and the choroid plexus provides a pathway for their entry into the central nervous system. These cytokines activate microglia, infiltrating immune cells (such as monocytes and B cells) and endothelial cells, ultimately exacerbating tissue damage (Castro et al. 2022).

## 5 Redox Signaling and Oxidative Stress

Maintaining a highly regulated mechanism to control intracellular reactive oxygen species (ROS) levels is crucial for sustaining normal cellular homeostasis (Holmström and Finkel 2014; Retta et al. 2012; Ushio‐Fukai 2009). ROS ensure appropriate endothelial responses to various stimuli and play a vital role in redox‐dependent regulation of numerous signaling processes (Retta et al. 2012). ROS play a pivotal role in orchestrating and modulating multiple redox‐sensitive signaling pathways and mechanisms to ensure that endothelial cells mount appropriate responses to diverse stimuli (Holmström and Finkel 2014; Retta et al. 2012; Ushio‐Fukai 2009). An imbalance between ROS production and clearance can lead to excessive ROS accumulation, triggering oxidative stress. This state can cause extensive oxidative damage to most cellular components, including proteins, lipids, and DNA (Zorov et al. 2014).

CCM genes (particularly KRIT1) play a major role in regulating cellular homeostasis and defense mechanisms against oxidative stress and inflammation. The loss of function of CCM genes (such as KRIT1) can produce pleiotropic downstream effects through the dysregulation of these key defense mechanisms, leading to disease onset (Retta and Glading 2016; Retta et al. 2020). KRIT1, with its pleiotropic functions, is associated with maintaining endothelial cell homeostasis and BBB integrity by controlling molecular and cellular responses to oxidative stress and inflammation (Goitre et al. 2014; Choquet et al. 2016; Antognelli et al. 2020). The loss of KRIT1 function has been shown to increase redox sensitivity, and pro‐oxidative and pro‐inflammatory states have been clearly identified as significant risk factors involved in the pathogenesis and progression of CCM (Vieceli et al. 2019; De Luca et al. 2021; Kim et al. 2020). The loss of KRIT1 function is associated with increased intracellular ROS levels and increased cellular susceptibility to oxidative stress‐mediated molecular and cellular dysfunction, involving the regulation of master regulators of the cellular response to oxidative stress (including FOXO1 and SOD2) in maintaining intracellular ROS homeostasis (Goitre et al. 2010). On the other hand, KRIT1 may exert its protective effects against oxidative stress by limiting pro‐oxidative and pro‐inflammatory pathways and mechanisms, including the redox‐sensitive JNK/c‐Jun/COX‐2 axis, the NADPH oxidase, and NF‐κB signaling (Goitre et al. 2014; Goitre et al. 2017). In mouse models of CCM disease, compounds with antioxidant properties can effectively rescue the major disease phenotypes associated with the loss of CCM gene function, including reduced endothelial barrier function and increased lesion burden (Goitre et al. 2010; Goitre et al. 2014; Gibson et al. 2015).

Deficiencies in CCM2 or CCM3 proteins have been shown to cause autophagic defects, mitochondrial dysfunction, and altered redox homeostasis, resembling the effects observed with KRIT1 deficiency (Marchi et al. 2015). CCM3 protects cells from apoptosis induced by oxidative stress and prevents CCM lesion formation by activating serine/threonine kinases of the germinal center kinase III (GCKIII) family, including MST4/STK26, MST3/STK24, and SOK1/STK25 (Fidalgo et al. 2012; Sartages et al. 2022).

Oxidative stress is a major inducer of vascular remodeling and dysfunction of the neurovascular unit (NVU) in cerebrovascular diseases and is closely related to the core molecular and cellular dysfunction associated with CCM (Chrissobolis et al. 2011; Freeman and Keller 2012; Faraci 2011). The CYP and MMP genes confer greater susceptibility to vascular diseases, are associated with oxidative stress, and are involved in regulating vascular homeostasis and remodeling (Niu and Qi 2012; Lee et al. 2007; Shahabi et al. 2014). In vascular tissues (including brain microvasculature), oxidative stress drives the expression and activity of MMPs, which can lead to oxidative stress‐mediated vascular dysfunction and breakdown of the BBB in cerebrovascular diseases (Spinale 2002; Chen et al. 2009). MMP enzymes mediate the proteolytic degradation of endothelial basement membrane components and the release of potentially bioactive molecules bound to the extracellular matrix (ECM), such as VEGF and TGF‐β1, playing a key role in inducing vascular remodeling and BBB disruption (Kelly et al. 2008; Haorah et al. 2007; Garcia‐Alloza et al. 2009).

In a chronic inflammatory microenvironment, the activation of microglia and excessive production of ROS promote the progression of CCM (Frias‐Anaya et al. 2024; Lo et al. 2022; Wang et al. 2015). The production of ROS may also be caused by the activation of immune cells and endothelial cells by pro‐inflammatory stimuli (Kim et al. 2013; Basuroy et al. 2009). Although not considered typical immunogenic cells, the increased innate immune response of endothelial cells has become an important mechanism in the interaction between oxidative stress, inflammation, and endothelial dysfunction (Chen et al. 2015).

## 6 Blood‐Brain Barrier Disruption Exacerbates Inflammation

The integrity of the blood‐brain barrier (BBB) relies on the concerted efforts of pericytes, microglia, astrocytes, and neurons (Retta et al. 2020; Freeman and Keller 2012; Bonkowski et al. 2011), which are also involved in leukocyte infiltration and local inflammatory responses (Dulamea and Lupescu 2024; Lai et al. 2022; Bonkowski et al. 2011).

During neuroinflammation, microglia play a crucial role by recognizing pathogen‐associated molecular patterns (PAMPs) such as lipopolysaccharide (LPS) through Toll‐like receptor 4 (TLR‐4), thereby strongly activating the innate immune response (Alam et al. 2019). This activation leads to a significant increase in the production of inflammatory mediators and cytokines (Alam et al. 2019). Activated microglia release various pro‐inflammatory mediators, including interleukin (IL)‐1β, IL‐6, tumor necrosis factor (TNF)‐α, and reactive oxygen species (ROS), which in turn induce neurotoxic effects (Wang et al. 2015; Orihuela et al. 2016). Moreover, the activation of microglia can induce the assembly of the NLRP3 inflammasome, a process that further promotes the maturation of the pro‐inflammatory cytokine IL‐1β (Tejera et al. 2019; Wang et al. 2019).

Pericytes, as important local regulatory cells, are involved in maintaining the BBB, vascular homeostasis, and hemostatic functions (Dore‐Duffy et al. 2006; Dai et al. 2022). Notably, increased pericyte coverage may also be an indicator of vascular dysfunction. In CCM3 knockout mouse models, pericytes exhibit reduced cellular extension, protrusion, and migration, along with diminished association with endothelial cells, reflecting the onset and progression of CCM (Wang et al. 2020).

These released cytokines have significant pro‐inflammatory functions: IL‐1β is involved in recruiting leukocytes to the site of inflammation and may be a key cytokine in regulating the transition from innate to adaptive immunity in the brain (Erta et al. 2012). TNF‐α, during neuroinflammation, may mediate the adhesion of T cells to brain endothelial cells (Dasgupta et al. 2007).

## 7 CCM Lesions Coexist With Thrombosis

Structural and functional changes in cerebral capillaries are the pathological hallmarks of CCM disease (Girouard et al. 2007). Each CCM lesion is marked by organized thrombi and leaking endothelium in the lumen of the vessel and hemolytic products in the surrounding brain parenchyma (Globisch et al. 2022). These lesions are often surrounded by encapsulated thrombus and thickened basement membranes, suggesting that intracerebral venous thrombosis may be an important factor in the development of CCM (Abe et al. 2005).

In CCM lesions, the balance of coagulation is severely disrupted. It has been found that procoagulant regions coexist with anticoagulant regions. This imbalance leads to loss of endothelial integrity and abnormal upregulation of both procoagulant and anticoagulant factors. Procoagulant structural domains leading to inflammation and disruption of endothelial integrity have been found to coexist with inflammatory responses induced by anticoagulant focal structural domains in CCM (Globisch et al. 2022, Lopez‐Ramirez et al. 2019). Dysfunctional endothelial cells in CCM lead to hemostatic deficits, resulting in the upregulation of both procoagulant and anticoagulant factors (Much et al. 2021). It is noteworthy that central nervous system hemorrhage has been associated with the presence of local anticoagulant endothelial receptors, such as the thrombomodulin (TM) and the endothelial protein C receptor (EPCR) expression (Lopez‐Ramirez et al. 2019). Dysfunctional endothelial cells also secrete a variety of cytokines (e.g., TNF‐α, IL‐1β), which promote T‐cell and neutrophil recruitment and adhesion and exacerbate inflammatory responses (Senden et al. 1998; Petri et al. 2010).

Thrombosis and procoagulant state within the lesion induce local hypoxia, which in turn stimulates the endothelial cells to produce nitric oxide (NO) and hypoxia‐inducible factor‐1α (HIF‐1α) (Lopez‐Ramirez et al. 2021; Globisch et al. 2022). Elevated levels of HIF‐1α induce astrocytes to upregulate the expression of vascular endothelial growth factor‐A (VEGF‐A), which is directly involved in blood‐brain barrier disruption (Lopez‐Ramirez et al. 2021; Argaw et al. 2009).

Tissue factor (TF) serves as the primary initiator of the coagulation cascade, which is produced by astrocytes (Eddleston et al. 1993); endothelial disruption further increases TF exposure (Grover and Mackman 2018). Also, astrocytes play an active role as immune effector cells by secreting proinflammatory factors, such as TNF‐α and IL‐1β (Chen et al. 2022). It has been shown that elevated levels of TNF‐α increase leukocyte migration across the endothelium, further contributing to the inflammatory milieu (Chandrasekharan et al. 2007). In addition, in a Ccm3 knockout mouse model, microglial cells showed a reactive morphology in thrombotic areas, and their degree of activation positively correlates with the number of thrombi around the lesion (Globisch et al. 2022). Finally, neutrophils are recruited into CCM lesions by releasing neutrophil extracellular traps (NETs), which act synergistically with coagulation factors to promote “immune thrombosis” (Yau et al. 2022; Yang et al. 2020).

Of note, pronounced astrogliosis and microgliosis accumulate around lesions (Gupta et al. 2010; Villanueva et al. 2011). Immunothrombotic lesions are enriched in neutrophils that undergo NETosis, releasing extracellular traps (NETs), which cooperate with coagulation factors to fuel ‘immunothrombosis’ (Gupta et al. 2019; Yang et al. 2019; Gupta et al. 2010; Villanueva et al. 2011; Grover and Mackman 2018; Tu et al. 2022; Mony et al. 2014). Non‐inflammatory thrombi aggregate components such as red blood cells, activated platelets, secreted von Willebrand factor (VWF), and fibrin clots (Globisch et al. 2022).

These findings suggest a complex interaction between proinflammatory cell and cytokine recruitment and procoagulant and anticoagulant structural domains within the CCM that synergistically promote CCM progression.

## 8 Discussion

CCM, as a complex vascular disease of the central nervous system, has a pathogenesis that involves multiple factors such as genetics, immunity, oxidative stress, and vascular dysfunction, which interact with each other to drive the disease onset and progression (Figures 1 and 2).

From a genetic perspective, the pathogenic basis of CCM is clear, and loss of function of the three major pathogenic genes, KRIT1 (CCM1), MGC4607 (CCM2), and PDCD10 (CCM3), is the key (Riolo et al. 2021; Fusco et al. 2022). However, gene mutations alone are not sufficient to drive the full progression of CCM, and synergistic effects of the second‐strike mechanism and microenvironmental stressors are indispensable. This suggests that although genetic factors provide the “soil” for the development of CCM, the actual manifestation and progression of the disease are also influenced by a variety of acquired factors, such as oxidative stress and inflammatory responses.

In terms of the inflammatory response, the infiltration of a large number of immune cells, such as macrophages, microglia, T cells, and B cells, in CCM lesions is closely associated with the aggressiveness of the lesion and the risk of recurrence (McDonald et al. 2011; Akers et al. 2009). B cells play a key role, with their clonal expansion and antigen‐driven maturation of their affinities contributing to the maturation and progression of the CCM lesion (Shi et al. 2009). In addition, the neutrophilic cells through the NETosis form extracellular traps that not only contribute directly to endothelial damage but also activate T/B cells that mediate adaptive immune responses (Yau et al. 2022; Villanueva et al. 2011; Dömer et al. 2021). The interplay of these inflammatory cells and factors creates a complex inflammatory network that exacerbates the pathologic process of CCM.

Dysregulation of the blood‐brain barrier further exacerbates the inflammatory response. Activation of microglia, release of proinflammatory cytokines, and pericyte abnormalities combine to disrupt the integrity of the blood‐brain barrier (Wang et al. 2015; Orihuela et al. 2016). This dysregulation not only promotes the infiltration of inflammatory cells but also leads to an imbalance in vascular homeostasis, which provides a favorable pathological environment for the progression of CCM.

Oxidative stress also plays an important role in the pathogenesis of CCM. Loss of function of CCM genes, such as KRIT1, leads to dysregulation of cellular defense mechanisms against oxidative stress, which in turn triggers oxidative stress (Goitre et al. 2014; Vieceli et al. 2019). Oxidative stress not only directly damages cellular components but also contributes to the inflammatory response and endothelial dysfunction through activation of various signaling pathways, such as the JNK/c‐Jun/COX‐2 axis, NADPH oxidase, and the NF ‐κB signaling pathways, among others, further exacerbating the inflammatory response and endothelial dysfunction (Goitre et al. 2014; Goitre et al. 2017). In addition, oxidative stress drives matrix metalloproteinase (MMP) expression and activity, leading to vascular remodeling and BBB rupture (Haorah et al. 2007).

In terms of vascular function, endothelial dysfunction in CCM lesions leads to an imbalance in hemostatic mechanisms, with the coexistence of procoagulant and anticoagulant structural domains. This imbalance not only triggers local hypoxia but also stimulates the production of NO and HIF‐1α by endothelial cells, which, in turn, induces astrocytes to upregulate the expression of VEGF‐A, further disrupting the BBB (Lopez‐Ramirez et al. 2021; Globisch et al. 2022). In addition, neutrophils act synergistically with coagulation factors through the release of NETs to promote “immune thrombosis,” further exacerbating the pathologic process of CCM (Olsson and Cedervall 2016; Villanueva et al. 2011; Dömer et al. 2021).

Although most studies support the crucial role of oxidative stress, inflammatory responses, and angiogenesis in the pathogenesis of CCM, some potential counterevidence exists (Shi et al. 2009; Kelly et al. 2008; Freeman and Keller 2012; Chen et al. 2015). Research has proposed the developmental venous anomaly (DVA) hypertension‐related ischemic or hemorrhagic vascular proliferation hypothesis, suggesting this may represent another significant factor in CCM development (Boulday et al. 2011; Bianconi et al. 2022). Increasing evidence indicates that aseptic inflammatory responses are key determinants in the formation and severity of CCM lesions. While heightened inflammatory responses and genetic expression polymorphisms are considered primary determinants of CCM pathogenesis and severity, these findings are primarily based on specific experimental models and populations, and their generalizability requires further validation.

When exploring the pathogenesis of CCM, researchers employ various experimental models and materials, which may exhibit significant differences depending on their sources and environments (Lopez‐Ramirez et al. 2019; Bianconi et al. 2022). Different cell models may differ in physiological function and gene expression, such as human umbilical vein endothelial cells (HUVECs). Furthermore, discrepancies exist between patient materials and mouse model tissues. The short growth cycle of mouse models may prevent them from fully simulating the complex pathological processes of human CCM.

In summarizing, the etiopathogenesis of CCM is a complex network of multifactorial and intertwined pathways. The pathological progression of CCM is driven by a complex interplay between vascular structural defects, imbalances in the coagulation‐anticoagulation system, inflammatory responses, and oxidative stress. Genetic factors provide the basis for the development of the disease, while factors such as oxidative stress, inflammatory response, and vascular dysfunction play a key role in driving the development and progression of the disease. The interaction between these factors not only exacerbates the aggressiveness of the lesions and the risk of recurrence but also leads to heterogeneity of clinical symptoms. Therefore, future research and treatment strategies need to take these factors into account with a view to finding more effective treatments and improving the prognosis of CCM patients.

## 9 Future Perspectives

Future research on CCM should focus on integrating multi‐omics approaches to better understand the complex interplay between genetic predispositions, immune microenvironment alterations, oxidative stress, and vascular dysfunction (Han et al. 2025; Tang et al. 2017). Given the heterogeneity of CCM, personalized treatment strategies based on molecular profiling of individual lesions will be crucial for improving patient outcomes. The development of standardized tools for assessing outcomes in CCM studies will enhance the comparability and reliability of research findings.

## Author Contributions

X. Z. conceived the idea for the paper and wrote the first version of the manuscript. X. X. and T. Y. provided mentorship and financial support. Y. Y. drafted the manuscript or revised it critically for important intellectual content. All authors read and approved the final version of the manuscript.

## Funding

This study was supported by the National Natural Science Foundation of China (No. 82401552) and the Shandong Provincial Natural Science Foundation (No. ZR2024QH590).

## Ethics Statement

No ethical support is required for this study.

## Conflicts of Interest

The authors declare no conflicts of interest.

## Data Availability

No data generated or analyzed during this study are included.
